# *rac*-7-[(2*E*)-But-2-eno­yl]-13-chloro-*N*-cyclo­hexyl-7,8-dihydro-5*H*-isochromeno[4,3-*c*]phenanthridine-8-carboxamide

**DOI:** 10.1107/S1600536812005946

**Published:** 2012-02-17

**Authors:** Ning Ye, Jin-Long Wu

**Affiliations:** a Laboratory of Asymmetric Catalysis and Synthesis, Department of Chemistry, Zhejiang University, Hangzhou, Zhejiang 310027, People’s Republic of China

## Abstract

In the title compound, C_31_H_29_ClN_2_O_3_, the two heterocyclic rings, belonging to a system of five condensed rings, adopt conformations intermediate between twist-boat and sofa. The secondary amide group is involved in a weak intramolecular N—H⋯N hydrogen bond. In the crystal, molecules are linked by pairs of C—H⋯Cl hydrogen bonds to form inversion dimers. These dimers are linked *via* a C—H⋯O interaction to form chains propagating along the *b*-axis direction.

## Related literature

For the Ugi four-component reaction of 2-amino­phenols, see: Xing *et al.* (2006 ▶); Dai *et al.* (2008 ▶). For microwave-assisted intra­molecular direct aryl­ation, see: Wu *et al.* (2007 ▶).
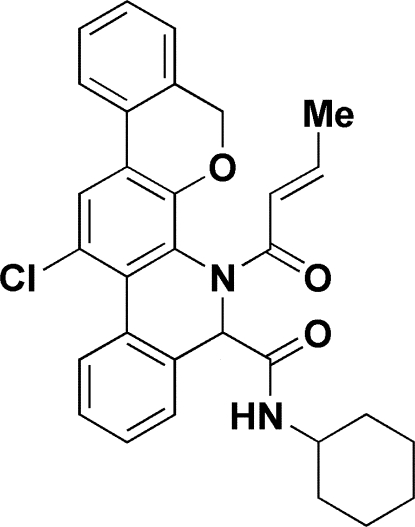


## Experimental

### 

#### Crystal data

C_31_H_29_ClN_2_O_3_
*M* *_r_* = 513.01
Monoclinic, 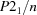

*a* = 13.2616 (8) Å
*b* = 13.4515 (7) Å
*c* = 14.8338 (9) Å
β = 95.067 (2)°
*V* = 2635.8 (3) Å^3^
*Z* = 4
Mo *K*α radiation
μ = 0.18 mm^−1^
*T* = 296 K
0.35 × 0.32 × 0.20 mm

#### Data collection

Rigaku RAXIS-RAPID/ZJUG diffractometer
Absorption correction: multi-scan (*ABSCOR*; Higashi, 1995 ▶) *T*_min_ = 0.930, *T*_max_ = 0.965
21259 measured reflections
4884 independent reflections
2797 reflections with *I* > 2σ(*I*)
*R*_int_ = 0.048

#### Refinement

*R*[*F* ^2^ > 2σ(*F* ^2^)] = 0.056
*wR*(*F* ^2^) = 0.197
*S* = 1.00
4884 reflections
336 parameters
H-atom parameters constrained
Δρ_max_ = 0.22 e Å^−3^
Δρ_min_ = −0.44 e Å^−3^

### 

Data collection: *PROCESS-AUTO* (Rigaku, 2006 ▶); cell refinement: *PROCESS-AUTO*; data reduction: *CrystalStructure* (Rigaku Americas and Rigaku, 2007 ▶); program(s) used to solve structure: *SHELXS97* (Sheldrick, 2008 ▶); program(s) used to refine structure: *SHELXL97* (Sheldrick, 2008 ▶); molecular graphics: *ORTEP-3 for Windows* (Farrugia, 1997 ▶); software used to prepare material for publication: *WinGX* (Farrugia, 1999 ▶).

## Supplementary Material

Crystal structure: contains datablock(s) I, global. DOI: 10.1107/S1600536812005946/gk2448sup1.cif

Structure factors: contains datablock(s) I. DOI: 10.1107/S1600536812005946/gk2448Isup2.hkl

Supplementary material file. DOI: 10.1107/S1600536812005946/gk2448Isup3.cml

Additional supplementary materials:  crystallographic information; 3D view; checkCIF report

## Figures and Tables

**Table 1:** Hydrogen-bond geometry (Å, °)

*D*—H⋯*A*	*D*—H	H⋯*A*	*D*⋯*A*	*D*—H⋯*A*
N2—H2⋯N1	0.86	2.41	2.758 (3)	105
C23—H23*B*⋯Cl1^i^	0.97	2.74	3.473 (4)	133
C14—H14⋯O3^ii^	0.93	2.58	3.391 (5)	146

Symmetry codes: (i) 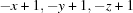; (ii) 
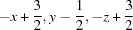
.

**Fig. 1.**
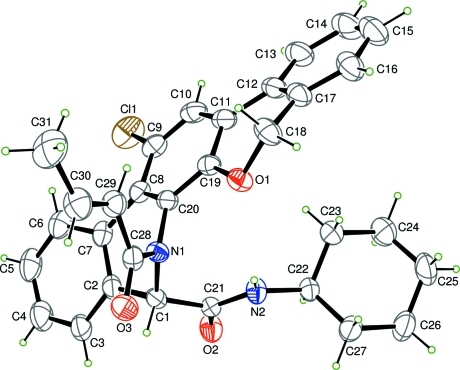
The molecular structure of the title compound with displacement ellipsoids drawn at the 40% probability level. H atoms are shown as a small spheres of arbitrary radius.

**Fig. 2.**
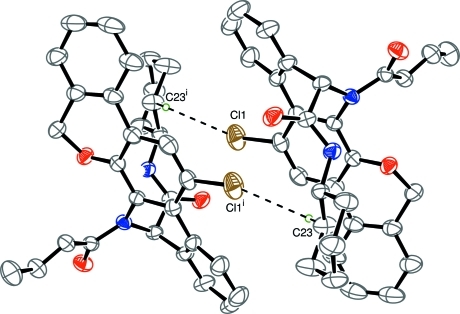
Centrosymmetric dimers by C—H···Cl hydrogen bonds. Symmetry code:(i) 1 - x, 1 - y, 1 - z.

## supplementary crystallographic information

### Comment

The title compound, C_31_H_29_ClN_2_O_3_, is a derivative of 7,8- dihydro-5*H*-6-oxa-7-azapicene, which was obtained from U-4CR of 2-amino-4-chlorophenol, 2-bromobenzaldehyde, (*E*)- crotonic acid and cyclohexyl isocyanide (Xing *et al.*, 2006; Dai *et al.*, 2008) followed by *O*-benzylation and palladium catalyzed intramolecular arylation (Wu *et al.*, 2007). The structure of the title compound has been characterized by spectroscopic methods with further confirmation by X-ray analysis. We report here its crystal structure.

In the molecule of the title compound (Fig. 1), there are three benzene rings and the middle one was fused with two other benzene rings by CH_2_O and CH_2_N bridges closing six-membered heterocyclic rings. The middle benzene ring is twisted relative to two other benzene rings by 30.7 (3)° and 15.7( 3)°. In the crystal structure, the molecules are linked by two C—H···Cl weak hydrogen bonds into centrosymmetric dimers (Fig. 2).

### Experimental

A solution of 2-amino-4-chlorophenol (3.0 mmol) and 2-bromobenzaldehyde (3.0 mmol) in MeOH (5 ml) was stirred at room temperature for 15 min. To the resultant mixture was added (*E*)-crotonic acid (3.0 mmol) followed by stirring for 5 min at the same temperature. Cyclohexyl isocyanide (3.0 mmol) was then added to the above mixture followed by stirring at 323 K for 48 h. The white precipitate of the U-4CR product was collected by filtration and the solid was washed with methanol (3 ml). The combined filtrate was concentrated under reduced pressure and the residue was purified by flash column chromatography over silica gel [eluting with 25% EtOAc in PE (b.p. 333–363 K)] to give additional portion of the U-4CR product. The yield of the U-4CR is 70%. A solution of the above U-4CR product (2.0 mmol), 2-bromobenzyl bromide (2.4 mmol), and K_2_CO_3_ (3.0 mmol) in acetone (reagent grade, 10 ml) was heated at 323 K for 2 h. The reaction was allowed to cool to room temperature. After adding water, the mixture was extracted using EtOAc (3 *x* 10 ml). The combined organic layer was dried over anhydrous Na_2_SO_4_, filtered off, and then evaporated under reduced pressure. The residue was purified by flash column chromatography over silica gel [eluting with 20% EtOAc in PE (b.p. 333–363 K)] to give the *O*-benzylation product (96%).

A 10-ml pressurized process vial was charged with the above *O*-benzylation product (0.15 mmol), Pd(OAc)_2_ (7.5 x 10^-3^ mmol; 5 mol %), K_2_CO_3_ (0.3 mmol), and PCy_3_HBF_4_ (1.5 x 10^-2^ mmol, 10 mol %). The vial was sealed with a cap containing a silicon septum. The vial was evacuated and backfilled with N_2_ (repeated for three times) through the cap using a needle. To the degassed vial was added degassed anhydrous MeCN (3 ml) through the cap using a syringe. The loaded vial was then placed into the microwave reactor cavity and was heated at 433 K for 80 min. After cooling to room temperature the reaction mixture was concentrated under reduced pressure and the residue was purified by flash column chromatography over silica gel [eluting with 25% EtOAc in PE (b.p. 333–363 K)] to give the title compound as a yellow solid (49 mg, 63%; m.p. 459–461 K (EtOAc-hexane). Single crystals suitable for X-ray diffraction were grown from a EtOAc/hexane mixture.

### Refinement

The H atoms were placed in calculated positions with C—H = 0.93–0.98 Å, N-H = 0.86 Å and included in the refinement as riding on their carrier atoms with *U*_iso_(H) =1.2*U*_eq_ (C,N).

### Crystal data

**Table:**

C_31_H_29_ClN_2_O_3_	*F*(000) = 1080
*M**_r_* = 513.01	*D*_x_ = 1.293 Mg m^−^^3^
Monoclinic, *P*2_1_/*n*	Mo *K*α radiation, λ = 0.71073 Å
Hall symbol: -P 2yn	Cell parameters from 12604 reflections
*a* = 13.2616 (8) Å	θ = 3.0–27.4°
*b* = 13.4515 (7) Å	µ = 0.18 mm^−^^1^
*c* = 14.8338 (9) Å	*T* = 296 K
β = 95.067 (2)°	Block, colorless
*V* = 2635.8 (3) Å^3^	0.35 × 0.32 × 0.20 mm
*Z* = 4	

### Data collection

**Table:**

Rigaku RAXIS-RAPID/ZJUG diffractometer	4884 independent reflections
Radiation source: rolling anode	2797 reflections with *I* > 2σ(*I*)
Graphite monochromator	*R*_int_ = 0.048
Detector resolution: 10.00 pixels mm^-1^	θ_max_ = 25.5°, θ_min_ = 3.0°
ω scans	*h* = −16→14
Absorption correction: multi-scan (*ABSCOR*; Higashi, 1995)	*k* = −16→16
*T*_min_ = 0.930, *T*_max_ = 0.965	*l* = −17→17
21259 measured reflections	

### Refinement

**Table:**

Refinement on *F*^2^	Secondary atom site location: difference Fourier map
Least-squares matrix: full	Hydrogen site location: inferred from neighbouring sites
*R*[*F*^2^ > 2σ(*F*^2^)] = 0.056	H-atom parameters constrained
*wR*(*F*^2^) = 0.197	*w* = 1/[σ^2^(*F*_o_^2^) + (0.095*P*)^2^ + 1.2622*P*] where *P* = (*F*_o_^2^ + 2*F*_c_^2^)/3
*S* = 1.00	(Δ/σ)_max_ < 0.001
4884 reflections	Δρ_max_ = 0.22 e Å^−^^3^
336 parameters	Δρ_min_ = −0.44 e Å^−^^3^
0 restraints	Extinction correction: *SHELXL*, Fc^*^=kFc[1+0.001xFc^2^λ^3^/sin(2θ)]^-1/4^
Primary atom site location: structure-invariant direct methods	Extinction coefficient: 0.0130 (19)

### Special details

**Table:**

Geometry. All e.s.d.'s (except the e.s.d. in the dihedral angle between two l.s. planes) are estimated using the full covariance matrix. The cell e.s.d.'s are taken into account individually in the estimation of e.s.d.'s in distances, angles and torsion angles; correlations between e.s.d.'s in cell parameters are only used when they are defined by crystal symmetry. An approximate (isotropic) treatment of cell e.s.d.'s is used for estimating e.s.d.'s involving l.s. planes.
Refinement. Refinement of *F*^2^ against ALL reflections. The weighted *R*-factor *wR* and goodness of fit *S* are based on *F*^2^, conventional *R*-factors *R* are based on *F*, with *F* set to zero for negative *F*^2^. The threshold expression of *F*^2^ > σ(*F*^2^) is used only for calculating *R*-factors(gt) *etc*. and is not relevant to the choice of reflections for refinement. *R*-factors based on *F*^2^ are statistically about twice as large as those based on *F*, and *R*- factors based on ALL data will be even larger.

### Fractional atomic coordinates and isotropic or equivalent isotropic displacement parameters (Å^2^)

**Table:**

	*x*	*y*	*z*	*U*_iso_*/*U*_eq_	
Cl1	0.57920 (9)	0.38756 (8)	0.47028 (9)	0.1040 (5)	
O1	0.79308 (16)	0.71162 (15)	0.66645 (13)	0.0592 (6)	
O2	0.51089 (18)	0.79850 (19)	0.41811 (15)	0.0731 (7)	
O3	0.88187 (18)	0.83063 (16)	0.43610 (16)	0.0692 (7)	
N1	0.76583 (18)	0.72488 (17)	0.48317 (15)	0.0485 (6)	
N2	0.60639 (19)	0.82566 (19)	0.54842 (16)	0.0576 (7)	
H2	0.6676	0.8320	0.5721	0.069*	
C1	0.6860 (2)	0.7620 (2)	0.41769 (19)	0.0525 (7)	
H1	0.7130	0.8192	0.3867	0.063*	
C2	0.6609 (2)	0.6817 (2)	0.3479 (2)	0.0589 (8)	
C3	0.6369 (3)	0.7041 (3)	0.2577 (2)	0.0764 (10)	
H3	0.6320	0.7701	0.2393	0.092*	
C4	0.6202 (3)	0.6291 (4)	0.1945 (3)	0.0940 (13)	
H4	0.6018	0.6446	0.1342	0.113*	
C5	0.6307 (3)	0.5322 (4)	0.2208 (3)	0.0940 (13)	
H5	0.6224	0.4820	0.1776	0.113*	
C6	0.6535 (3)	0.5080 (3)	0.3105 (3)	0.0770 (10)	
H6	0.6603	0.4415	0.3273	0.092*	
C7	0.6663 (2)	0.5820 (2)	0.3765 (2)	0.0593 (8)	
C8	0.6843 (2)	0.5634 (2)	0.4748 (2)	0.0543 (8)	
C9	0.6473 (3)	0.4825 (2)	0.5230 (3)	0.0641 (9)	
C10	0.6594 (3)	0.4779 (2)	0.6151 (3)	0.0675 (9)	
H10	0.6339	0.4231	0.6439	0.081*	
C11	0.7086 (2)	0.5524 (2)	0.6681 (2)	0.0591 (8)	
C12	0.7110 (3)	0.5590 (3)	0.7674 (2)	0.0653 (9)	
C13	0.6507 (3)	0.4996 (3)	0.8181 (3)	0.0873 (12)	
H13	0.6072	0.4525	0.7899	0.105*	
C14	0.6565 (4)	0.5118 (4)	0.9117 (3)	0.1064 (17)	
H14	0.6177	0.4715	0.9460	0.128*	
C15	0.7185 (4)	0.5825 (5)	0.9541 (3)	0.1045 (16)	
H15	0.7215	0.5896	1.0166	0.125*	
C16	0.7759 (3)	0.6425 (4)	0.9043 (2)	0.0876 (12)	
H16	0.8166	0.6915	0.9328	0.105*	
C17	0.7733 (3)	0.6302 (3)	0.8105 (2)	0.0670 (9)	
C18	0.8411 (3)	0.6891 (3)	0.7556 (2)	0.0725 (10)	
H18A	0.8593	0.7507	0.7869	0.087*	
H18B	0.9028	0.6519	0.7496	0.087*	
C19	0.7487 (2)	0.6314 (2)	0.6215 (2)	0.0504 (7)	
C20	0.7366 (2)	0.6362 (2)	0.52781 (19)	0.0488 (7)	
C21	0.5920 (2)	0.7966 (2)	0.46243 (19)	0.0505 (7)	
C22	0.5240 (2)	0.8474 (2)	0.60503 (19)	0.0533 (7)	
H22	0.4599	0.8336	0.5692	0.064*	
C23	0.5303 (3)	0.7807 (3)	0.6868 (2)	0.0775 (11)	
H23A	0.5934	0.7928	0.7234	0.093*	
H23B	0.5295	0.7118	0.6676	0.093*	
C24	0.4415 (4)	0.7998 (3)	0.7433 (3)	0.1050 (15)	
H24A	0.3787	0.7830	0.7081	0.126*	
H24B	0.4478	0.7575	0.7964	0.126*	
C25	0.4383 (4)	0.9064 (3)	0.7721 (3)	0.0954 (14)	
H25A	0.4975	0.9211	0.8131	0.114*	
H25B	0.3788	0.9174	0.8044	0.114*	
C26	0.4355 (3)	0.9754 (3)	0.6917 (3)	0.0875 (12)	
H26A	0.4392	1.0437	0.7127	0.105*	
H26B	0.3719	0.9669	0.6549	0.105*	
C27	0.5232 (3)	0.9550 (3)	0.6338 (3)	0.0753 (10)	
H27A	0.5167	0.9973	0.5806	0.090*	
H27B	0.5868	0.9710	0.6682	0.090*	
C28	0.8642 (2)	0.7546 (2)	0.4774 (2)	0.0519 (7)	
C29	0.9452 (2)	0.6895 (2)	0.5181 (2)	0.0622 (8)	
H29	0.9285	0.6381	0.5560	0.075*	
C30	1.0403 (3)	0.7021 (3)	0.5023 (3)	0.0793 (11)	
H30	1.0556	0.7577	0.4688	0.095*	
C31	1.1257 (3)	0.6351 (4)	0.5333 (3)	0.1114 (16)	
H31A	1.1018	0.5839	0.5711	0.167*	
H31B	1.1778	0.6728	0.5670	0.167*	
H31C	1.1527	0.6054	0.4817	0.167*	

### Atomic displacement parameters (Å^2^)

**Table:**

	*U*^11^	*U*^22^	*U*^33^	*U*^12^	*U*^13^	*U*^23^
Cl1	0.0962 (8)	0.0717 (6)	0.1430 (11)	−0.0234 (5)	0.0050 (7)	−0.0170 (6)
O1	0.0654 (14)	0.0622 (13)	0.0484 (12)	−0.0042 (10)	−0.0033 (10)	0.0042 (10)
O2	0.0611 (15)	0.1040 (18)	0.0528 (13)	0.0172 (13)	−0.0030 (12)	−0.0067 (12)
O3	0.0746 (16)	0.0604 (13)	0.0752 (15)	−0.0084 (11)	0.0224 (12)	0.0076 (12)
N1	0.0487 (14)	0.0486 (13)	0.0488 (13)	−0.0027 (11)	0.0070 (11)	0.0074 (11)
N2	0.0509 (15)	0.0712 (16)	0.0511 (15)	0.0036 (12)	0.0063 (12)	−0.0106 (12)
C1	0.0565 (18)	0.0570 (17)	0.0444 (16)	0.0068 (14)	0.0072 (14)	0.0064 (13)
C2	0.0571 (19)	0.074 (2)	0.0464 (17)	0.0115 (16)	0.0094 (15)	−0.0041 (15)
C3	0.088 (3)	0.092 (3)	0.0493 (19)	0.023 (2)	0.0087 (18)	−0.0014 (19)
C4	0.102 (3)	0.125 (4)	0.054 (2)	0.020 (3)	0.002 (2)	−0.019 (2)
C5	0.089 (3)	0.119 (4)	0.072 (3)	0.011 (3)	0.001 (2)	−0.041 (3)
C6	0.074 (2)	0.081 (2)	0.075 (3)	0.0063 (19)	0.003 (2)	−0.023 (2)
C7	0.0512 (18)	0.072 (2)	0.0546 (18)	0.0052 (15)	0.0040 (15)	−0.0145 (16)
C8	0.0501 (18)	0.0527 (16)	0.0602 (19)	0.0050 (14)	0.0056 (15)	−0.0017 (14)
C9	0.058 (2)	0.0506 (17)	0.084 (3)	−0.0041 (14)	0.0056 (18)	−0.0002 (17)
C10	0.063 (2)	0.0535 (18)	0.087 (3)	−0.0015 (15)	0.0115 (19)	0.0172 (18)
C11	0.0567 (19)	0.0575 (18)	0.064 (2)	0.0049 (15)	0.0082 (16)	0.0153 (16)
C12	0.061 (2)	0.075 (2)	0.061 (2)	0.0131 (17)	0.0134 (17)	0.0249 (17)
C13	0.084 (3)	0.097 (3)	0.085 (3)	0.009 (2)	0.028 (2)	0.034 (2)
C14	0.096 (4)	0.138 (4)	0.092 (3)	0.022 (3)	0.044 (3)	0.053 (3)
C15	0.088 (3)	0.160 (5)	0.069 (3)	0.031 (3)	0.028 (3)	0.035 (3)
C16	0.073 (3)	0.130 (3)	0.060 (2)	0.021 (2)	0.0050 (19)	0.014 (2)
C17	0.059 (2)	0.088 (2)	0.0541 (19)	0.0130 (19)	0.0061 (16)	0.0145 (18)
C18	0.068 (2)	0.099 (3)	0.0489 (19)	−0.0030 (19)	−0.0044 (16)	0.0071 (18)
C19	0.0463 (17)	0.0498 (16)	0.0552 (18)	0.0016 (13)	0.0042 (14)	0.0070 (14)
C20	0.0494 (17)	0.0472 (15)	0.0502 (17)	0.0017 (13)	0.0074 (13)	0.0045 (13)
C21	0.058 (2)	0.0492 (16)	0.0444 (16)	0.0078 (14)	0.0065 (15)	0.0014 (13)
C22	0.0527 (18)	0.0602 (17)	0.0476 (16)	0.0029 (14)	0.0087 (14)	−0.0040 (14)
C23	0.098 (3)	0.070 (2)	0.067 (2)	0.006 (2)	0.024 (2)	0.0056 (18)
C24	0.132 (4)	0.099 (3)	0.093 (3)	0.006 (3)	0.059 (3)	0.014 (2)
C25	0.104 (3)	0.117 (3)	0.070 (2)	0.009 (3)	0.039 (2)	−0.012 (2)
C26	0.094 (3)	0.080 (2)	0.092 (3)	0.016 (2)	0.030 (2)	−0.017 (2)
C27	0.091 (3)	0.060 (2)	0.079 (2)	0.0059 (18)	0.031 (2)	−0.0018 (17)
C28	0.0542 (18)	0.0525 (16)	0.0506 (17)	−0.0058 (14)	0.0136 (14)	−0.0058 (14)
C29	0.0525 (19)	0.069 (2)	0.066 (2)	−0.0002 (15)	0.0118 (16)	0.0002 (16)
C30	0.056 (2)	0.099 (3)	0.084 (3)	−0.0011 (19)	0.0132 (19)	−0.001 (2)
C31	0.059 (3)	0.141 (4)	0.134 (4)	0.021 (3)	0.007 (3)	0.000 (3)

### Geometric parameters (Å, º)

**Table:**

Cl1—C9	1.713 (3)	C14—C15	1.373 (7)
O1—C19	1.373 (3)	C14—H14	0.9300
O1—C18	1.448 (4)	C15—C16	1.370 (6)
O2—C21	1.211 (4)	C15—H15	0.9300
O3—C28	1.225 (3)	C16—C17	1.397 (5)
N1—C28	1.374 (4)	C16—H16	0.9300
N1—C20	1.434 (3)	C17—C18	1.492 (5)
N1—C1	1.460 (4)	C18—H18A	0.9700
N2—C21	1.332 (4)	C18—H18B	0.9700
N2—C22	1.465 (3)	C19—C20	1.387 (4)
N2—H2	0.8600	C22—C23	1.506 (4)
C1—C2	1.513 (4)	C22—C27	1.510 (4)
C1—C21	1.535 (4)	C22—H22	0.9800
C1—H1	0.9800	C23—C24	1.525 (5)
C2—C3	1.382 (4)	C23—H23A	0.9700
C2—C7	1.407 (4)	C23—H23B	0.9700
C3—C4	1.381 (5)	C24—C25	1.498 (6)
C3—H3	0.9300	C24—H24A	0.9700
C4—C5	1.363 (6)	C24—H24B	0.9700
C4—H4	0.9300	C25—C26	1.510 (5)
C5—C6	1.378 (6)	C25—H25A	0.9700
C5—H5	0.9300	C25—H25B	0.9700
C6—C7	1.396 (4)	C26—C27	1.530 (5)
C6—H6	0.9300	C26—H26A	0.9700
C7—C8	1.478 (4)	C26—H26B	0.9700
C8—C20	1.401 (4)	C27—H27A	0.9700
C8—C9	1.413 (4)	C27—H27B	0.9700
C9—C10	1.364 (5)	C28—C29	1.473 (4)
C10—C11	1.399 (5)	C29—C30	1.315 (4)
C10—H10	0.9300	C29—H29	0.9300
C11—C19	1.397 (4)	C30—C31	1.488 (6)
C11—C12	1.474 (5)	C30—H30	0.9300
C12—C17	1.384 (5)	C31—H31A	0.9600
C12—C13	1.398 (5)	C31—H31B	0.9600
C13—C14	1.392 (6)	C31—H31C	0.9600
C13—H13	0.9300		
			
C19—O1—C18	114.5 (2)	O1—C18—H18B	109.2
C28—N1—C20	124.6 (2)	C17—C18—H18B	109.2
C28—N1—C1	119.7 (2)	H18A—C18—H18B	107.9
C20—N1—C1	112.5 (2)	O1—C19—C20	117.1 (2)
C21—N2—C22	123.9 (3)	O1—C19—C11	121.6 (3)
C21—N2—H2	118.1	C20—C19—C11	121.0 (3)
C22—N2—H2	118.1	C19—C20—C8	122.5 (3)
N1—C1—C2	108.3 (2)	C19—C20—N1	119.3 (3)
N1—C1—C21	112.6 (2)	C8—C20—N1	117.6 (3)
C2—C1—C21	112.0 (3)	O2—C21—N2	124.0 (3)
N1—C1—H1	107.9	O2—C21—C1	119.2 (3)
C2—C1—H1	107.9	N2—C21—C1	116.8 (3)
C21—C1—H1	107.9	N2—C22—C23	110.6 (3)
C3—C2—C7	120.1 (3)	N2—C22—C27	112.2 (3)
C3—C2—C1	121.7 (3)	C23—C22—C27	110.2 (3)
C7—C2—C1	118.2 (3)	N2—C22—H22	107.9
C4—C3—C2	120.5 (4)	C23—C22—H22	107.9
C4—C3—H3	119.8	C27—C22—H22	107.9
C2—C3—H3	119.8	C22—C23—C24	110.6 (3)
C5—C4—C3	119.9 (4)	C22—C23—H23A	109.5
C5—C4—H4	120.1	C24—C23—H23A	109.5
C3—C4—H4	120.1	C22—C23—H23B	109.5
C4—C5—C6	120.7 (4)	C24—C23—H23B	109.5
C4—C5—H5	119.7	H23A—C23—H23B	108.1
C6—C5—H5	119.7	C25—C24—C23	111.2 (4)
C5—C6—C7	120.8 (4)	C25—C24—H24A	109.4
C5—C6—H6	119.6	C23—C24—H24A	109.4
C7—C6—H6	119.6	C25—C24—H24B	109.4
C6—C7—C2	117.9 (3)	C23—C24—H24B	109.4
C6—C7—C8	124.8 (3)	H24A—C24—H24B	108.0
C2—C7—C8	117.3 (3)	C24—C25—C26	111.2 (3)
C20—C8—C9	115.6 (3)	C24—C25—H25A	109.4
C20—C8—C7	117.7 (3)	C26—C25—H25A	109.4
C9—C8—C7	126.5 (3)	C24—C25—H25B	109.4
C10—C9—C8	121.8 (3)	C26—C25—H25B	109.4
C10—C9—Cl1	115.7 (3)	H25A—C25—H25B	108.0
C8—C9—Cl1	122.4 (3)	C25—C26—C27	111.5 (3)
C9—C10—C11	122.5 (3)	C25—C26—H26A	109.3
C9—C10—H10	118.8	C27—C26—H26A	109.3
C11—C10—H10	118.8	C25—C26—H26B	109.3
C19—C11—C10	116.5 (3)	C27—C26—H26B	109.3
C19—C11—C12	118.2 (3)	H26A—C26—H26B	108.0
C10—C11—C12	124.9 (3)	C22—C27—C26	110.8 (3)
C17—C12—C13	119.6 (3)	C22—C27—H27A	109.5
C17—C12—C11	117.6 (3)	C26—C27—H27A	109.5
C13—C12—C11	122.8 (4)	C22—C27—H27B	109.5
C14—C13—C12	119.0 (5)	C26—C27—H27B	109.5
C14—C13—H13	120.5	H27A—C27—H27B	108.1
C12—C13—H13	120.5	O3—C28—N1	119.9 (3)
C15—C14—C13	121.1 (4)	O3—C28—C29	122.4 (3)
C15—C14—H14	119.4	N1—C28—C29	117.6 (3)
C13—C14—H14	119.4	C30—C29—C28	121.8 (3)
C16—C15—C14	120.0 (4)	C30—C29—H29	119.1
C16—C15—H15	120.0	C28—C29—H29	119.1
C14—C15—H15	120.0	C29—C30—C31	125.7 (4)
C15—C16—C17	120.1 (5)	C29—C30—H30	117.1
C15—C16—H16	120.0	C31—C30—H30	117.1
C17—C16—H16	120.0	C30—C31—H31A	109.5
C12—C17—C16	120.2 (3)	C30—C31—H31B	109.5
C12—C17—C18	118.6 (3)	H31A—C31—H31B	109.5
C16—C17—C18	121.1 (4)	C30—C31—H31C	109.5
O1—C18—C17	111.9 (3)	H31A—C31—H31C	109.5
O1—C18—H18A	109.2	H31B—C31—H31C	109.5
C17—C18—H18A	109.2		
			
C28—N1—C1—C2	101.4 (3)	C15—C16—C17—C18	−175.2 (4)
C20—N1—C1—C2	−59.1 (3)	C19—O1—C18—C17	−48.5 (4)
C28—N1—C1—C21	−134.2 (3)	C12—C17—C18—O1	36.9 (4)
C20—N1—C1—C21	65.2 (3)	C16—C17—C18—O1	−146.4 (3)
N1—C1—C2—C3	−143.1 (3)	C18—O1—C19—C20	−155.8 (3)
C21—C1—C2—C3	92.2 (4)	C18—O1—C19—C11	30.0 (4)
N1—C1—C2—C7	34.5 (4)	C10—C11—C19—O1	176.0 (3)
C21—C1—C2—C7	−90.3 (3)	C12—C11—C19—O1	2.7 (4)
C7—C2—C3—C4	−1.4 (5)	C10—C11—C19—C20	2.1 (4)
C1—C2—C3—C4	176.1 (3)	C12—C11—C19—C20	−171.2 (3)
C2—C3—C4—C5	−2.2 (6)	O1—C19—C20—C8	−174.1 (3)
C3—C4—C5—C6	2.9 (7)	C11—C19—C20—C8	0.1 (4)
C4—C5—C6—C7	0.0 (6)	O1—C19—C20—N1	−3.0 (4)
C5—C6—C7—C2	−3.5 (5)	C11—C19—C20—N1	171.1 (3)
C5—C6—C7—C8	176.0 (3)	C9—C8—C20—C19	−2.0 (4)
C3—C2—C7—C6	4.2 (5)	C7—C8—C20—C19	173.2 (3)
C1—C2—C7—C6	−173.4 (3)	C9—C8—C20—N1	−173.3 (3)
C3—C2—C7—C8	−175.3 (3)	C7—C8—C20—N1	2.0 (4)
C1—C2—C7—C8	7.1 (4)	C28—N1—C20—C19	71.5 (4)
C6—C7—C8—C20	153.5 (3)	C1—N1—C20—C19	−129.1 (3)
C2—C7—C8—C20	−27.0 (4)	C28—N1—C20—C8	−117.0 (3)
C6—C7—C8—C9	−31.8 (5)	C1—N1—C20—C8	42.4 (3)
C2—C7—C8—C9	147.6 (3)	C22—N2—C21—O2	10.9 (5)
C20—C8—C9—C10	1.9 (5)	C22—N2—C21—C1	−171.1 (3)
C7—C8—C9—C10	−172.9 (3)	N1—C1—C21—O2	−156.4 (3)
C20—C8—C9—Cl1	178.5 (2)	C2—C1—C21—O2	−34.0 (4)
C7—C8—C9—Cl1	3.8 (5)	N1—C1—C21—N2	25.5 (4)
C8—C9—C10—C11	0.2 (5)	C2—C1—C21—N2	147.8 (3)
Cl1—C9—C10—C11	−176.7 (3)	C21—N2—C22—C23	120.5 (3)
C9—C10—C11—C19	−2.2 (5)	C21—N2—C22—C27	−116.1 (3)
C9—C10—C11—C12	170.6 (3)	N2—C22—C23—C24	−177.2 (3)
C19—C11—C12—C17	−15.1 (4)	C27—C22—C23—C24	58.2 (4)
C10—C11—C12—C17	172.2 (3)	C22—C23—C24—C25	−57.6 (5)
C19—C11—C12—C13	162.3 (3)	C23—C24—C25—C26	55.3 (6)
C10—C11—C12—C13	−10.3 (5)	C24—C25—C26—C27	−54.3 (5)
C17—C12—C13—C14	−1.6 (6)	N2—C22—C27—C26	179.2 (3)
C11—C12—C13—C14	−178.9 (3)	C23—C22—C27—C26	−57.0 (4)
C12—C13—C14—C15	1.5 (7)	C25—C26—C27—C22	55.2 (5)
C13—C14—C15—C16	0.1 (7)	C20—N1—C28—O3	178.4 (3)
C14—C15—C16—C17	−1.6 (7)	C1—N1—C28—O3	20.4 (4)
C13—C12—C17—C16	0.1 (5)	C20—N1—C28—C29	1.5 (4)
C11—C12—C17—C16	177.6 (3)	C1—N1—C28—C29	−156.5 (3)
C13—C12—C17—C18	176.9 (3)	O3—C28—C29—C30	−9.2 (5)
C11—C12—C17—C18	−5.6 (5)	N1—C28—C29—C30	167.6 (3)
C15—C16—C17—C12	1.5 (6)	C28—C29—C30—C31	−174.1 (4)

### Hydrogen-bond geometry (Å, º)

**Table:**

*D*—H···*A*	*D*—H	H···*A*	*D*···*A*	*D*—H···*A*
N2—H2···N1	0.86	2.41	2.758 (3)	105
C23—H23*B*···Cl1^i^	0.97	2.74	3.473 (4)	133
C14—H14···O3^ii^	0.93	2.58	3.391 (5)	146

Symmetry codes: (i) −*x*+1, −*y*+1, −*z*+1; (ii) −*x*+3/2, *y*−1/2, −*z*+3/2.
